# Relationship between Thyroid Feedback Quantile‐based Index and cardiovascular diseases in a population with normal thyroid function: Evidence from the National Health and Nutrition Examination Survey 2007–2012

**DOI:** 10.1002/clc.24271

**Published:** 2024-04-28

**Authors:** Hui Li, Xue Liu, Xinhui Wang, Qingqing Yang

**Affiliations:** ^1^ Jinan University, Guangdong Guangzhou China; ^2^ Department of Cardiology The First Affiliated Hospital of Bengbu Medical College Bengbu Anhui Province China; ^3^ Department of Endocrinology, Shandong Provincial Hospital, Cheeloo College Of Medicine Shandong University Jinan Shandong China; ^4^ Department of Endocrinology The First Affiliated Hospital of Bengbu Medical College Bengbu Anhui Province China

**Keywords:** cardiovascular disease, NHANES, TFQI, thyroid

## Abstract

**Background:**

Previous study has demonstrated a link between TFQI, indicating the central sensitivity of thyroid hormones, and conditions like obesity, diabetes, and metabolic syndrome.

**Hypothesis:**

Nevertheless, the potential relationship between TFQI and cardiovascular disease (CVD) in individuals with normal thyroid function has yet to be established.

**Methods:**

The present research is a retrospective cohort investigation that included a total of 6297 individuals who had normal function of the thyroid and no history of thyroid disorders. These participants were selected from National Health and Nutrition Examination Survey data set, covering the years 2007–2012. The calculation of TFQI was performed depending on FT4 and TSH. Given the complex survey design and sample weights, we used multivariate linear regression models and stratified analysis to evaluate TFQI's correlation with CVD.

**Results:**

Subjects with CVD had greater levels of TFQI than those with no CVD. After adjusting for other covariates, TFQI exhibited a positive association with CVD risk, and the OR was 1.706 (*p* = .005). In subgroup analyses that were stratified by sex and BMI, it was shown that female individuals who had CVD had greater levels of TFQI in comparison to female participants without CVD (*p* = .002). Furthermore, elevated levels of TFQI were consistently connected to a raised incidence of CVD in the BMI (>28 kg/m^2^) group after regulating for different covariates. Furthermore, correlation analysis showed an association between TFQI and metabolic biomarkers.

**Conclusions:**

The levels of TFQI are strongly connected to the prevalence of CVD, indicating that energy metabolism may be related to the occurrence of CVD.

## Graphical abstract

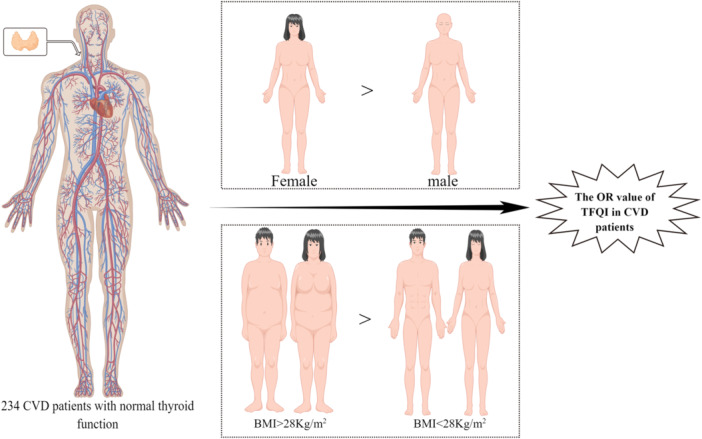

## 1 INTRODUCTION

Cardiovascular disease (CVD) is a prevalent reason for mortality and disability globally.^1^ It encompasses a range of diseases that influence the heart and blood vessels. The major kinds of CVDs are heart failure, stroke, coronary heart disease (CHD), and angina.^2^ CVDs have a complex pathogenesis, and their development is often influenced by various risk factors like elevated systolic blood pressure, smoking, high blood glucose, increased LDL cholesterol, and obesity/overweight.

Thyroid hormones are crucial in regulating energy metabolism and are strongly linked to metabolic disturbances, even within the normal range.^3^ They have diverse effects on the cardiovascular system, including influencing endothelial cell function, blood pressure, and myocardial oxygen consumption.^4^ Moreover, they are associated with common risk factors for CVD.^5^, ^6^ The relationship between CVD and thyroid dysfunction, whether hyperthyroidism or hypothyroidism, has been extensively studied.^7^ However, research is scarce on CVD in subjects with normal function of the thyroid. Nonetheless, even in persons with normal function of the thyroid, thyroid hormones are closely connected to metabolic disturbances.^8^, ^9^ Even slight fluctuations in thyroid hormone concentrations can contribute to an increased incidence and mortality rate of CVD.^10^, ^11^

The sensitivity index of thyroid hormone reveals the dynamic association between thyroid hormones and TSH in individuals with normal function of thyroid. The set point of the feedback loop in the central nervous system can be influenced by phenomena of central resistance, while phenomena of peripheral resistance can diminish the metabolic impacts of hormones. A commonly used indicator to examine the sensitivity index of peripheral thyroid hormone is the free triiodothyronine (FT3)/free thyroxine (FT4) ratio, which reveals the overall balance and sensitivity of the thyroid hormone system. Investigations have indicated a frequent decrease in the FT3/FT4 ratio in individuals with CVD.^12^, ^13^, ^14^ For instance, in subjects with normal function of the thyroid, a significant negative association has been observed between the FT3/FT4 ratio and the occurrence of CHD.^12^ A reduced FT3/FT4 ratio is closely linked with adverse outcomes in cases of nonobstructive coronary arteries^13^ and acute myocardial infarction in subjects with type 2 diabetes.^14^

However, there have been limited reports regarding the link between the central thyroid hormone sensitivity index and CVD. A recently recognized novel index of thyroid hormone resistance is called the Thyroid Feedback Quantile‐based Index (TFQI). TFQI allows for evaluating the relation between normal function of the thyroid and conditions like diabetes, obesity, and metabolic syndrome.^3^ TFQI is derived from the empirical joint distribution of FT4 and thyroid‐stimulating hormone (TSH). One advantage of TFQI is its ability to avoid extreme values in cases of thyroid dysfunction, thereby providing greater stability compared to other sensitivity indices of central thyroid hormone, including the Thyrotroph T4 Resistance Index (TT4RI) and the TSH Index (TSHI).

Here, we aimed to assess whether TFQI is linked to CVD in individuals with normal function of the thyroid. To accomplish this objective, we used the comprehensive data system of the National Health and Nutrition Examination Survey (NHANES). This investigation focused on examining the correlation between TFQI and overall cardiovascular events.

## 2 METHODS

### 2.1 Study population

**Figure 1:**
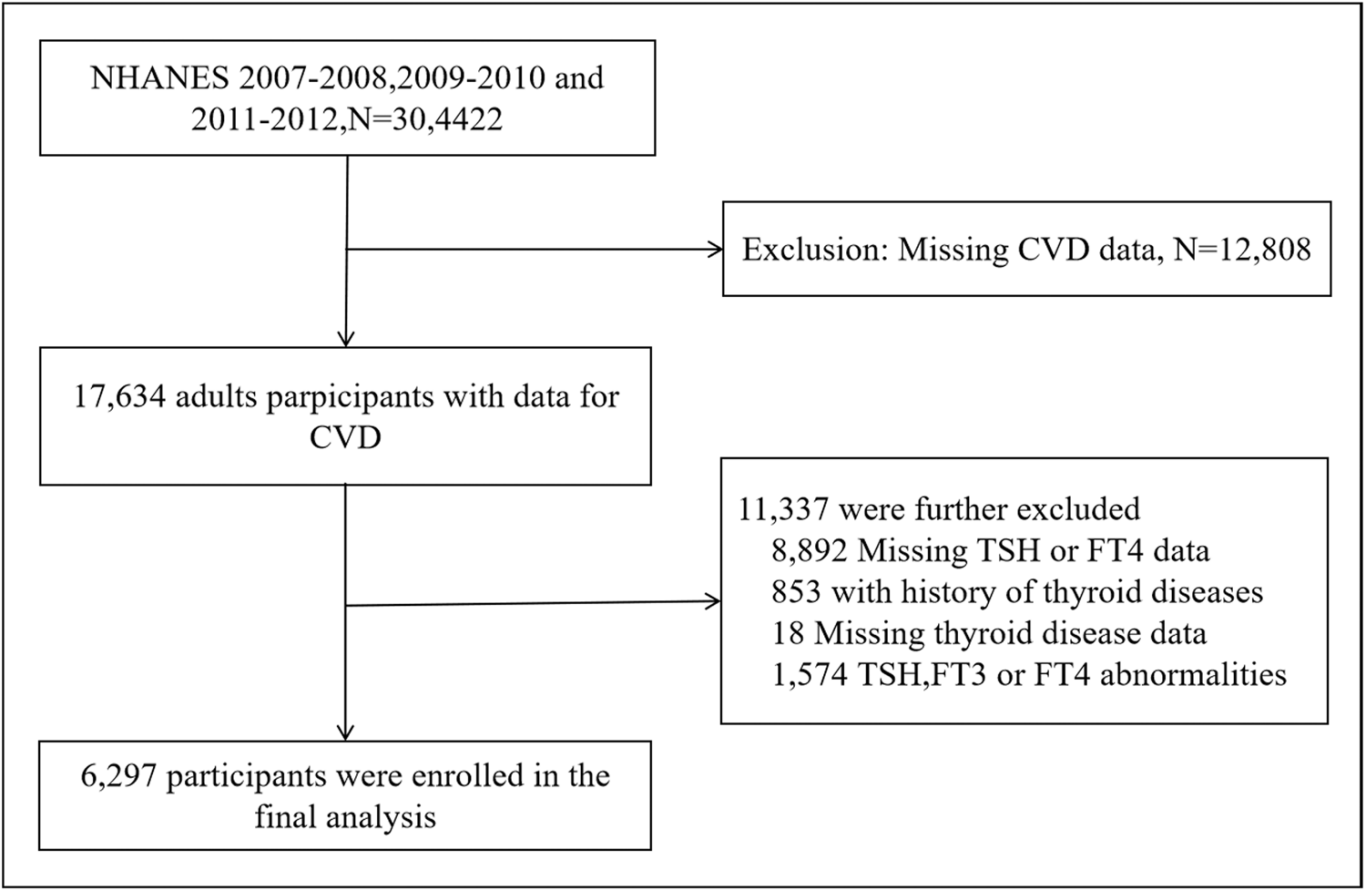
Flow diagram of study participants.

The NHANES survey is a continuous investigation performed by the Centers for Disease Control and Prevention (CDC) and managed by the National Center for Health Statistics (NCHS).^15^ The survey provides a nationally representative sample of noninstitutionalized people in the United States. The survey collects data from approximately 5000 adults and releases new data every 2 years. To ensure the accuracy of the information, we utilized a complex, stratified, multiple‐phase probability cluster sampling design. Participants in the survey provided information on various aspects, including general health information, demographic, socioeconomic, and dietary (via 24‐h dietary recall). The survey follows ethical guidelines, and all participants or their proxies give written informed consent. The investigation procedure was authorized by the Institutional Review Board of NCHS, CDC. For our analysis, we used data from NHANES for 2007–2008, 2009–2010, and 2011–2012. The total sample size consisted of 6297 individuals who were 19 years old or older. To ensure the reliability of our findings, we excluded cases with a thyroid disorder history, those with abnormal values for FT3, FT4, or TSH, and those with missing data. This exclusion allowed us to minimize the potential impact of medicines related to thyroid gland disease on function and autoimmunity of thyroid (Figure 1).

### 2.2 Definitions of CVD

A questionnaire of standardized medical conditions was conducted in individual interviews to obtain CVD diagnoses. The participants were queried on whether they had ever received information about certain illnesses from a doctor or other healthcare professional. To identify participants with CVD, we classified individuals as having a CVD if they reported any of the following conditions: congestive heart failure (CHF), myocardial infarction (MI), CHD, angina pectoris, and stroke. The reported results were then converted into a binary variable to indicate the presence or absence of CVD.

### 2.3 Determination of serum thyroid function

The present study investigated thyroid parameters, including FT4, FT3, and TSH. Specimens were collected and processed following the guidelines defined in the NHANES Laboratory/Medical Technologists Procedures Manual (LPM). A third‐generation, two‐site immunoenzymatic (“sandwich”) assay was employed with a 0.34–5.6 µIU/mL reference range to measure TSH levels. A competitive binding immunoenzymatic assay was utilized with a reference range of 2.5–3.9 pg/mL to determine FT3 levels. A two‐step enzyme immunoassay was utilized with a reference range of 7.74–20.64 pmol/L to detect FT4 levels. In the present investigation, those who exhibited TSH, FT3, and FT4 values that were within the established reference range were classified as euthyroid (EU), demonstrating the normal function of thyroid.

### 2.4 Calculation of TFQI

Based on the empirical cumulative distribution function, TFQI was detected using the values of FT4 and TSH. For instance, in the case of a CVD patient, the FT4 value was 0.7, and the TSH value was 1.128. Using the cdf function from the R programming package, we calculated the cdf(FT4) and cdf(TSH) to be 0.2825155 and 0.2926791, respectively. Subsequently, we used the formula TFQI = cdf(FT4)–(1–cdf(TSH)), which yielded a TFQI value of −0.424805. The TFQI has the benefit of preventing the presence of outliers in thyroid dysfunction cases while also exhibiting more stability compared to other indices like TT4RI and TSHI. The value of TFQI ranges from −1 to 1. Prior investigations have shown that a negative TFQI value indicates higher hypothalamic‐pituitary‐thyroid (HPT) axis sensitivity to the changes in FT4 levels. Conversely, a positive TFQI value suggests reduced HPT axis sensitivity to FT4. A TFQI value of 0 reveals a normal HPT axis sensitivity to alterations in FT4 levels.^9^

### 2.5 Covariates of interest

The covariates in this study include laboratory, demographic, and questionnaire data. Demographic data consists of age, sex, and level of education. Systolic and diastolic blood pressure (SBP and DBP) were detected in all groups. Laboratory data includes fasting glucose (FPG) in mmol/l, AST (U/L), HbA1c in percentage (%), ALT (U/L), uric acid (UA) in mg/dL, creatinine in umol/L, triglycerides (TG) in mg/dL, total cholesterol (TC) in mmol/L, HDL‐cholesterol (HDL‐c) in mmol/L, LDL‐cholesterol (LDL‐c) in mmol/L, and C‐reactive protein (CRP) in mg/dL. The questionnaire data includes WC (waist circumference) in cm, BMI (body mass index) in kg/m^2^, presence of diabetes, hyperlipidemia, and hypertension.

### 2.6 Statistical analysis

This study used SPSS 26 (IBM SPSS Statistics) and R 4.2.1 (R Foundation for Statistical Computing) program for data analysis and chart creation. A significance level of *p* < .05 was reflected as statistically significant unless otherwise specified. The analysis of normally or skewed distributed data is presented as mean ± standard deviation, while categorical variables are presented as numbers and percentages (%). The *χ* ^2^ test was employed for categorical variables. Kruskal–Wallis test was utilized for skewed continuous variables, and One‐way analysis of variance (ANOVA) was performed for normally distributed continuous variables. Binary logistic regression analysis was used to explore the possible associations between TFQI and CVD, diabetes, dyslipidemia, and hypertension. The study calculated the odds ratios (OR) for TFQI increase and the corresponding 95% confidence intervals (CI) per standard deviation (SD). Model 1 was unadjusted, Model 2 included adjustments for age and gender, and Model 3 adjusted for education level, hypertension, diabetes, hypercholesterolemia, UA, CRP, BMI, and FT3. Collinearity tests verified the lack of multicollinearity among the independent variables. To improve the coherence of the data, subgroup analyses were conducted, stratifying the participants based on gender and BMI. Spearman correlation analysis was used to assess the relationship between TFQI and common metabolic indicators. Missing values were addressed using multiple imputations to avoid potential survival bias. Sensitivity analysis was conducted to assess the statistical insignificance of the differences in each indicator before and after imputation.

## 3 RESULTS

### 3.1 Participant features

Table 1 displays the fundamental features of subjects categorized according to quartiles of serum TFQI ratio. Our investigation included 6297 participants, of which 234 participants had CVD. The average subjects' age was 48.76 (17.51) years, and 52.36% were male. Subjects with higher TFQI values tended to be older, male, and more educated. Furthermore, significant variations were observed between the four groups regarding the occurrence of diabetes, hypertension, and hyperlipidemia and levels of blood glucose, HbA1c, HDL‐c, UA, CRP, and BMI (*p* < .05). Furthermore, among CVD patients, FT4, and TSH levels were significantly higher (*p* < .05).

**Table 1:** Baseline characteristics of the research population with and without CVD.

	Total	Quartile 1 (≤−0.19)	Quartile 2 (−0.18 to 0.07)	Quartile 3 (0.18–0.34)	Quartile 4 (≥0.35–0.19)	*p* Value
Participants, no	6297	1580	1545	1596	1576	
age (years)	48.76 ± 17.51	44.67 ± 15.58	47.47 ± 17.36	50.16 ± 17.74	52.71 ± 18.23	<.001
Gender (%)						.189
Male	3,297 (52.36)	822 (52.03)	787 (50.94)	827 (51.82)	861 (54.63)	
Female	3,000 (47.64)	758 (47.97)	758 (49.06)	769 (48.18)	715 (45.37)	
Edu (%)						.006
Less than high school	1799 (28.6)	425 (26.9)	434 (28.0)	479 (30.0)	461 (29.3)	
High school	1448 (23.0)	421 (26.6)	350 (22.7)	346 (21.7)	331 (21.0)	
More than high school	3050 (48.4)	734 (46.5)	761 (49.3)	771 (48.3)	784 (49.7)	
Hyperlipidemia (%)	4,577 (72.69)	1,114 (70.51)	1,094 (70.81)	1,185 (74.25)	1,184 (75.13)	
Diabetic (%)	1,017 (16.15)	177 (11.20)	234 (15.15)	289 (18.11)	317 (20.11)	<.001
Hypertension (%)	2,517 (39.97)	553 (35.00)	569 (36.83)	670 (41.98)	725 (46.00)	<.001
FPG (mmol/l)	6.14 ± 2.08	5.93 ± 1.73	6.06 ± 1.86	6.23 ± 2.21	6.35 ± 2.43	<.001
HbA1c (%)	5.75 ± 1.09	5.62 ± 0.92	5.7 ± 0.99	5.81 ± 1.16	5.87 ± 1.24	<.001
Alt (U/L)	25.91 ± 24.02	26.13 ± 18.33	25.65 ± 27.51	25.49 ± 19.37	26.38 ± 29.12	.834
Ast (U/L)	26.21 ± 19.73	26.41 ± 16.12	26.32 ± 28.84	25.81 ± 13.43	26.3 ± 17.26	.708
UA (mg/dL)	327.32 ± 86.4	318.42 ± 85.31	324.03 ± 86.9	330.55 ± 82.99	336.18 ± 89.37	<.001
TG (mg/dL)	2.22 ± 2.31	2.27 ± 2.5	2.22 ± 2.28	2.2 ± 2.25	2.17 ± 2.18	.220
TC (mmol/l)	5.05 ± 1.06	5.06 ± 1.09	5.05 ± 1.03	5.06 ± 1.08	5.03 ± 1.06	.503
HDL‐c (mmol/l)	1.34 ± 0.4	1.37 ± 0.41	1.34 ± 0.39	1.33 ± 0.4	1.32 ± 0.38	.002
LDL‐c (mmol/l)	2.72 ± 0.98	2.69 ± 1.01	2.71 ± 0.96	2.73 ± 0.98	2.73 ± 0.96	.180
CRP (mg/dL)	0.43 ± 0.81	0.36 ± 0.68	0.41 ± 0.71	0.43 ± 0.73	0.53 ± 1.05	<.001
BMI (kg/m^2^)	28.83 ± 6.69	28.05 ± 5.97	28.73 ± 6.4	29.13 ± 6.99	29.42 ± 7.25	<.001
FT3 (pg/mL)	3.17 ± 0.3	3.17 ± 0.29	3.16 ± 0.29	3.16 ± 0.29	3.17 ± 0.31	.620
FT4 (pmol/l)	10.47 ± 1.44	9.39 ± 0.69	10.25 ± 1.26	10.66 ± 1.45	11.57 ± 1.3	<.001
TSH (µIU/mL)	1.75 ± 0.95	1.01 ± 0.38	1.41 ± 0.63	1.99 ± 0.97	2.57 ± 0.85	<.001
CVD (%)	234 (3.72)	38 (2.41)	45 (2.91)	62 (3.88)	89 (5.65)	<.001

*Note*: Mean ± SD for continuous variables: The *p* Value was calculated by the weighted linear regression model. (%) for categorical variables: The *p* Value was calculated by the weighted *χ* ^2^ test.

Abbreviation: CVD, cardiovascular disease.

### 3.2 Relations between TFQI and the presence of CVD, diabetes, hyperlipidemia, and hypertension

A logistic analysis was accomplished to assess the link between TFQI and CVD, diabetes, hyperlipidemia, and hypertension. The results revealed that TFQI was positively connected with CVD, diabetes, hyperlipidemia, and hypertension risks (*p* < .05). However, after regulating many covariates, TFQI was not found to be significantly associated with hyperlipidemia and hypertension. Nevertheless, the positive relationship between TFQI and the occurrence of CVD and diabetes was still significant in Models 2 and 3. Specifically, each 1‐unit rise in TFQI was connected to a 70.6% elevation in the CVD risk in the multivariable Model 3 (OR = 1.706, 95% CI: 1.173–2.481, *p* = .005), and each 1‐unit rise in TFQI was linked with a 42.2% elevation in the diabetes risk in the multivariable Model 3 (OR = 1.422, 95% CI: 1.159–1.744, *p* = .001; Table 2). Additionally, we incorporated SBP, DBP, FPG, HbA1c, ALT, AST, creatinine, UA, TG, TC, HDL‐c, LDL‐c, waist circumference, and BMI to formulate a multiple logistic regression equation. The findings unveiled that TFQI exerts a statistically significant influence on CVD (OR = 1.724, 95% CI 1.19–2.50, *p* = .004)(Supporting Information).

**Table 2:** Associations between TFQI and the presence of CVD, diabetes, hyperlipidemia, and hypertension.

	Model 1, OR (95% CI, *p* Value)	Model 2, OR (95% CI, *p* Value)	Model 3, OR (95% CI, *p* Value)
CVD	2.552 (1.793, 3.633) <.001	1.864 (1.297, 2.681) .001	1.706 (1.173, 2.481) .005
Diabetic^a^	2.033 (1.697, 2.435) <.001	1.529 (1.266, 1.847) <.001	1.422 (1.159, 1.744) .001
Hyperlipidemia^b^	1.266 (1.093, 1.465) .002	1.024 (0.880, 1.193) .757	0.940 (0.802, 1.101) .442
Hypertension^c^	1.636 (1.431, 1.872) <.001	1.169 (1.005, 1.356) .043	1.029 (0.879, 1.205) .723

*Note*: Logistic regression models. Model 1: No covariates were adjusted. Model 2 was adjusted for age (two groups) and gender. Model 3 was adjusted for age (two groups), gender, education, diabetes, hypertension, hyperlipidemia, UA, CRP, BMI, and FT3.

Abbreviations: CRP, C‐reactive protein; CVD, cardiovascular disease.

a In Model 3, the inclusion of covariates excluded diabetes as a risk factor.

^b^ In Model 3, the inclusion of covariates excluded hyperlipidemia as a risk factor.

^c^ In Model 3, the inclusion of covariates excluded hypertension as a risk factor.

### 3.3 Subgroup analyses stratified by gender and BMI

Table 3 displays the subgroup analyses that have been stratified based on sex and BMI. Individuals with elevated levels of TFQI had a greater prevalence of CVD in contrast to those with lower levels, regardless of sex (OR = 2.019, 95% CI: 1.344–3.033, *p* = .001 for males; OR = 4.875, 95% CI: 2.350–10.114, *p* < .001 for females). After regulating for several covariates, TFQI remained significantly associated with CVD in females (OR = 3.375, 95% CI: 1.564–7.280, *p* = .002).

**Table 3:** Subgroup analyses stratified by gender and BMI.

	Model 1, OR (95% CI, *p* Value)	Model 2, OR (95% CI, *p* Value)	Model 3, OR (95% CI, *p* Value)
TFQI	2.552 (1.793, 3.633) <.001	1.864 (1.297, 2.681) .001	1.706 (1.173, 2.481) .005
Subgroup analysis stratifed by gender			
Male	2.019 (1.344, 3.033) .001	1.560 (1.025, 2.375) .038	1.376 (0.889, 2.128) .152
Female	4.875 (2.350, 10.114) <.001	3.334 (1.595, 6.967) .001	3.375 (1.564, 7.280) .002
Subgroup analysis stratifed by BMI			
BMI < 18.5	0.133 (0.004, 4.424) .259	0.136 (0.004, 4.427) .262	0.017 (0.000, 13.646) .234
BMI 18.5–23.9	2.641 (1.101, 6.335) .030	1.735 (0.731, 4.121) .211	2.207 (0.867, 5.619) .097
BMI 24–28	3.714 (1.862, 7.409) <.001	2.497 (1.229, 5.072) .011	1.903 (0.922, 3.928) .082
BMI > 28	2.336 (1.447, 3.772) .001	1.873 (1.136, 3.087) .014	1.707 (1.018, 2.862) .043

*Note*: Logistic regression models: Model 1: No covariates were adjusted. Model 2 was adjusted for age (two groups) and gender. Model 3 was adjusted for age (two groups), gender, education, diabetes, hypertension, hyperlipidemia, UA, CRP, BMI, and FT3.

Abbreviations: CRP, C‐reactive protein; UA, uric acid.

Additionally, after regulating for different covariates, we observed that greater TFQI levels were consistently connected with a raised CVD risk in the BMI (>28 kg/m^2^) group. However, no significant relationship was detected between TFQI and CVD in other BMI groups.

### 3.4 Correlation analysis of TFQI and metabolic indexes

The correlation analysis reveals the connection between TFQI and metabolic indicators. TFQI had a positive association with HbA1c (*r* = .082, *p* < .001), FPG (*r* = .074, *p* < .001), and UA levels (*r* = .077, *p* < .001). Conversely, TFQI exhibited a negative connection with HDL‐C (*r* = −0.036, *p* = .004) levels (Table 4).

**Table 4:** Correlation analysis of TFQI and metabolic indexes.

Item	HbA1c	FPG	UA	TG	TC	HDL‐C	LDL‐C
TFQI							
r	.082	.074	.077	.011	−.012	−.036	.013
*p* Value	<.001	<.001	<.001	.381	.323	.004	.315

Abbreviations: TC, total cholesterol; TG, triglyceride; UA, uric acid.

## 4 DISCUSSION

This investigation examined the association between a central thyroid hormone sensitivity index, TFQI, and the prevalence of CVD based on the NHANES 2007–2008, 2009–2010, and 2011–2012 data. Our results demonstrated a strong correlation between TFQI and the incidence of CVD. The greater TFQI levels were linked with raised CVD occurrence. The strongest association between TFQI and the prevalence of CVD was seen in the subgroup analyses that were stratified by gender and BMI. Specifically, this link was most prominent in the female subgroup and among those with a BMI of more than 28 kg/m^2^.

Over the years, significant attention has been given to the effect of hyperthyroidism or hypothyroidism on CVD. However, recent research has suggested that even in individuals with normal function of the thyroid, higher levels of thyroid hormone resistance index are associated with an increased incidence and adverse prognosis of CVD.^12^, ^13^, ^16^, ^17^ Furthermore, there is a greater incidence of diabetes, obesity, and metabolic syndrome in individuals with elevated thyroid hormone resistance index.^18^, ^19^ Our study aligns with these prior findings, as we found a positive connection between TFQI and the incidence of CVD and a strong association with diabetes. The primary pathological mechanism underlying the development of CVD is atherosclerosis, a condition characterized by plaque buildup in the arteries. Previous research has established that high blood glucose levels, hypertension, and dyslipidemia are risk factors for atherosclerosis development in diabetic individuals.^20^ Individuals with thyroid hormone resistance may exhibit elevated levels of circulating cholesterol and triglycerides, decreased levels of HDL‐C, and decreased systemic insulin sensitivity.^21^ Our study performed a correlation analysis to investigate the relationship between TFQI and various metabolic markers. TFQI was positively correlated with HbA1c, FPG, and UA, whereas TFQI was negatively correlated with HDL‐C. The outcomes of this study demonstrate that the use of TFQI can serve as a beneficial method for detecting minor acquired thyroid hormone resistance in the general population. This condition is often linked to energy imbalance.

Research has shown that TSHI and TFQI levels are greater in obese cases compared with the control group, and after weight loss surgery, TSHI and TFQI significantly decrease over time.^22^ Additionally, a cross‐sectional investigation revealed a significant negative connection between thyroid secretion capacity (SPINA‐GT) and TSHI with obesity indices, revealing that obesity diminishes the levels of thyroid hormone sensitivity indices.^23^ Our study also confirmed that even after regulating several factors, TFQI remains a CVD prevalence risk factor in obese people.

Physiologically, there is a negative connection between thyroid hormones and TSH because of the negative feedback loop. However, in individuals with thyroid hormone resistance syndrome, a condition inherited as an autosomal dominant trait, high levels of thyroid hormones coexist with high TSH levels.^24^, ^25^ Additionally, a proposed reversible form of acquired thyroid hormone resistance may occur because of internal compensatory mechanisms.^26^ Prolonged fasting can cause a reduction in TSH levels and a rise in the sensitivity of the pituitary gland to thyroid hormones.^27^, ^28^ Moreover, individuals with morbid obesity often exhibit greater thyroid hormones and TSH levels.^29^ The clinical presentations of thyroid hormone resistance may be classified into two main categories: central resistance, which impacts the feedback loop set point of the central nervous system, and peripheral resistance, which diminishes the metabolic actions of thyroid hormones. Hence, the use of the thyroid hormone resistance index may be advantageous in the observation and assessment of new CVD treatments that target energy expenditure.

Decreased thyroid hormone sensitivity has been connected to elevated levels of residual cholesterol in euthyroid adults. Studies have reported that thyroid hormone receptor α (TRα) has a vital function in preventing the development of atherosclerosis by inhibiting apoptosis in vascular smooth muscle cells (VSMCs) and reducing the production of inflammatory cytokines in macrophages.^30^, ^31^ Recent research has indicated that TRα1, when triggered by various T3 or T4 levels, effectively suppresses apoptosis in macrophages treated with oxidized low‐density lipoprotein (oxLDL), providing new insights into the antiatherosclerotic effects of thyroid hormones.^32^ Additionally, individuals in the euthyroid population who exhibit thyroid hormone insensitivity are at an increased risk of experiencing additional cardiac metabolic risks.^33^ Previous studies have also highlighted the effect of hypothyroidism. Hypothyroidism can trigger apoptosis in VSMCs, myocardial cells, and hematopoietic progenitor cells. Furthermore, thyroid hormones have been found to reduce the rate of apoptosis in these cells dose‐dependently.^30^, ^34^, ^35^ Another potential connection between the function of the thyroid and the cardiovascular system is the modulation of circadian rhythms.^36^ The presence of chronic disturbances in circadian rhythms has been linked to a raised risk of obesity, diabetes, and CVD, hence promoting the existing evidence on the correlation between thyroid dysfunction and the risk of cardiovascular complications. These findings emphasize the thyroid hormone's function in preserving the integrity and function of cardiovascular tissues, as disturbances in thyroid function can lead to cellular apoptosis and potentially contribute to CVD.

## 5 LIMITATIONS

The present study involved a relatively small sample size of CVD patients, and the correlation analysis only provided a preliminary analysis of the trends in two variables. Further studies must confirm the relationship between TFQI and metabolic indicators in a huge population by considering age, disease duration, and gender to eliminate potential confounders.

## 6 CONCLUSION

In the normal thyroid function people in the United States, TFQI was found to be an independent predictor of CVD after multiple adjustments. TFQI was also correlated with metabolic indicators. Therefore, it is essential to pay equal attention to TFQI in the general population, as TFQI can assist in early prevention and treatment of coronary heart disease.

## CONFLICT OF INTEREST STATEMENT

The authors declare no conflict of interest.

## Supporting information

Supporting information.

## Data Availability

The data that support the findings of this study are available from the corresponding author.
