# Supplementary material for: Relationship between Thyroid Feedback Quantile‐based Index and cardiovascular diseases in a population with normal thyroid function: Evidence from the National Health and Nutrition Examination Survey 2007–2012
Source: Clin Cardiol. 2024 Apr 28;47(5):e24271. doi: 10.1002/clc.24271 (PMC11056699; doi:10.1002/clc.24271)
Supplement: Supplementary file 1 — Supporting information. [file CLC-47-e24271-s001.doc]

Logistic Regression Analysis Results of the Association Between TFQI and CVD Risk.

| Evaluation Metrics | OR（95%CI） | *P* |
| --- | --- | --- |
| SBP(mmHg) | 1.015(1.008,1.023) | <0.001 |
| DBP(mmHg) | 0.988(0.977,0.999) | 0.031 |
| FPG (mmol/l) | 0.975(0.894,1.064) | 0.571 |
| HbA1c (%) | 1.349(1.141,1.597) | <0.001 |
| Alt (U/L) | 1.003(0.995,1.010) | 0.517 |
| Ast (U/L) | 1.000(0.989,1.011) | 0.968 |
| Creatinine (umol/L) | 1.724(1.397,2.126) | <0.001 |
| UA (mg/dL) | 1.001(0.999,1.003) | 0.262 |
| TG (mg/dL) | 0.897(0.577,1.395) | 0.629 |
| TC (mmol/l) | 0.752(0.268,2.108) | 0.588 |
| HDL-c (mmol/l) | 0.548(0.181,1.658) | 0.287 |
| LDL-c (mmol/l) | 0.717(0.253,2.034) | 0.532 |
| WC (cm) | 1.082(1.060,1.105) | <0.001 |
| BMI (kg/m2) | 0.834(0.790,0.879) | <0.001 |
| TFQI | 1.724(1.187,2.502) | 0.004 |
